# Fear of hypoglycemia—An underestimated problem

**DOI:** 10.1002/brb3.2633

**Published:** 2022-05-27

**Authors:** Agnieszka Przezak, Weronika Bielka, Piotr Molęda

**Affiliations:** ^1^ Department of Diabetology and Internal Medicine Pomeranian Medical University Szczecin Poland

**Keywords:** anxiety, diabetes, fear of hypoglycemia, hypoglycemia

## Abstract

**Introduction:**

Fear of hypoglycemia (FOH) is a phenomenon that affects people with diabetes experiencing hypoglycemia. On the one hand, FOH is an adaptive mechanism that helps to protect patients from hypoglycemia and its consequences. On the other hand, the non‐normative level of FOH causes anxiety and tension, disturbs normal functioning, and makes normoglycemia maintenance difficult.

**Objective:**

The main objective of this review was to describe factors influencing FOH and methods of measurement of FOH levels. Moreover, we highlighted the impact of the new technologies used in diabetes therapy on FOH and different therapeutic possibilities helping patients cope with excessive levels of FOH. We also presented clinical cases of patients with high FOH levels met in clinical practice and discussed methods to better diagnose and assist people with this kind of problem.

**Methods:**

We searched for studies and articles via PubMed using the keywords fear of hypoglycemia, diabetes, and hypoglycemia. From screened documents identified from literature search, 67 articles were included in our review.

**Results:**

We divided results from literature screening into five parts: fear of hypoglycemia and hypoglycemia definition, risk factors for the FOH, methods of measuring levels of FOH, therapies for the FOH, and modern technologies. We also described clinical examples of abnormal fear of hypoglycemia in patients.

**Conclusion:**

The review highlights the importance of taking into consideration fear of hypoglycemia phenomenon in diabetic patients in everyday clinical practice.

## Graphical abstract

Fear of hypoglycemia is a phenomenon that affects people with diabetes experiencing hypoglycemia. On the one hand, fear of hypoglycemia is an adaptive mechanism that helps to protect patients from hypoglycemia and its consequences. On the other hand, the non‐normative level of fear of hypoglycemia causes anxiety and tension, disturbs normal functioning, and makes normoglycemia maintenance difficult.

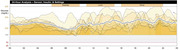

## 1 INTRODUCTION

Anxiety disorders are mental disorders that are not caused by organic injury to the central nervous system, and their symptoms relate mainly to emotional processes. These disorders might be rooted in past experiences that trigger anxiety despite the absence of actual threat at a certain moment (Kępiński, 2020). It is an anticipatory reaction to a threatening stimulus that may arise for no apparent reason (Wredling et al., 1992). In anxiety disorders, the subjective level of anxiety and its frequency disorganize the patient's life and cause suffering (LeDoux, 2017). Anxiety disorders and hypoglycemia have a similar clinical manifestation, and the accompanying symptoms can be categorized into those resulting from the stimulation of the autonomic nervous system (e.g., arrhythmias, hyperhidrosis) and neurological symptoms (e.g., cognitive impairment or behavioral disturbances) (Kępiński, 2020; Szadkowska, 2012). For this reason, anxiety might mask the symptoms of hypoglycemia (Boyle et al., 2004).

Recurrent hypoglycemia may result in the fear of hypoglycemia (FOH) (Wredling et al., 1992). It is a state of unpleasant tension, anxiety, and discomfort, manifested by, among other things, palpitations, shortness of breath, or hand tremors, which are present in many diabetic patients who experience hypoglycemia or are at risk of developing it (Krawczyk et al., 2020). A high level of FOH in patients might lead to behaviors inappropriate to the actual risk of hypoglycemia, suboptimal metabolic control of diabetes, and a significant reduction in the patient's quality of life (Böhme et al., 2013). This problem is often underestimated in everyday clinical practice.

## 2 Hypoglycemia

### 2.1 Definition

Hypoglycemia is diagnosed when the blood glucose level drops below 3.9 mmol/L (70 mg/dl) regardless of clinical symptoms (Araszkiewicz et al., 2021). Symptoms of hypoglycemia also may occur with higher blood glucose levels, especially if a rapid decrease in blood glucose has occurred (Szadkowska, 2012). Clinically significant hypoglycemia is diagnosed when the blood glucose level is lower than 3.0 mmol/L (54 mg/dl). Severe hypoglycemia is an episode requiring the help of another person, regardless of blood glucose level (Araszkiewicz et al., 2021). Table 1 presents the categories of hypoglycemia according to the American Diabetes Association (2021).

**TABLE 1:** Categories of hypoglycemia according to the American Diabetes Association (202^1^)

Level of hypoglycemia	Criteria/description
Level 1	Blood glucose value between 3.0 mmol/L (54 mg/dl) and 3.9 mmol/L (70 mg/dl)
Level 2	Blood glucose level lower than 3.0 mmol/L (54 mg/dl)
Level 3	Severe hypoglycemic events characterized by altered mental and/or physical status that require assistance for resolution

Hypoglycemia is the most common acute complication of diabetes treatment (Szadkowska, 2012). It is a consequence of the pursuit of optimal blood glucose control to reduce the risk of long‐term complications in diabetes (Duckworth et al., 2009; Inzucchi et al., 2012; Nathan et al., 1993; Patel et al., 2008). It concerns all types of diabetes, particularly patients with type 1 diabetes. The global Hypoglycemia Assessment Tool study in approximately 28,000 people from 24 countries revealed that patients with type 1 diabetes had on average 73 episodes of hypoglycemia per year, including 11 nocturnal episodes and five severe episodes, while patients with type 2 diabetes treated with insulin had 19, four, and approximately three episodes per year, respectively. Large variations in hypoglycemia rates among countries were also observed. The highest rates of any hypoglycemia were observed in Latin America for type 1 diabetes and in Russia for type 2 diabetes (Khunti et al., 2016). Recurrent hypoglycemic episodes deteriorate well‐being and quality of life of patients with diabetes (Polonsky et al., 2018). Hypoglycemic episodes are unpleasant and also create a potential threat to patients’ health and life. The symptoms of hypoglycemia change with blood glucose levels. Initial symptoms include a feeling of hunger, excessive sweating, pallor, palpitations, headache and dizziness, and other ailments associated with stimulation of the autonomic nervous system. Further symptoms developing because of insufficient glucose supply to the central nervous system include behavioral disorders, cognitive disorders, consciousness disorders, seizures, or loss of consciousness. Prolonged severe hypoglycemia might ultimately lead to death (Szadkowska, 2012).

The risk of hypoglycemia depends on the type of antihyperglycemic therapy. The risk is higher in patients treated with insulin as well as sulfonylureas and glinides that stimulate the secretion of endogenous insulin (Balijepalli et al., 2017). The main reasons for the occurrence of hypoglycemia are the failure to adjust the insulin dose to food intake or physical activity (Cryer, 2013). Liver insufficiency, renal failure, hypopituitarism, hypothyroidism, or adrenal cortex insufficiency coexisting with diabetes also contribute to an excessive reduction in blood glucose levels (Kalra et al., 2013). Complications of hypoglycemia include traumas, cardiovascular events, and progressive dementia (Amiel, 2021).

Recurrent hypoglycemic episodes may lead to hypoglycemia unawareness, when the patient does not experience symptoms of hypoglycemia despite low blood glucose levels. Therefore, patients have more frequent and longer episodes of hypoglycemia, which they are unaware of (Vignesh & Mohan, 2004). Recurrent episodes of hypoglycemia also influence the occurrence and intensification of FOH (Wredling et al., 1992).

### 2.2 Fear of hypoglycemia

Depending on its intensity, the FOH might be normal or abnormal. Normal fear, otherwise known as adaptive, allows the patient to respond adequately to the risk of hypoglycemia. It is an evolutionarily developed defense mechanism against anticipated danger. On the other hand, abnormal fear is persistent, recurrent, or objectively inappropriate to the risk of hypoglycemia, and its intensity might be too low or too high (Óhman, 2005). Low levels of fear cause the disregard of risk, underestimation of symptoms, and creation opportunities for subsequent episodes of hypoglycemia, which increases the risk of potentially life‐threatening hypoglycemia or its complications. On the other hand, excessively high fear causes constant anxiety, emotional stress, discomfort, and insecurity, which in turn leads to a significant reduction in the self‐assessed quality of life in patients with diabetes and may trigger or intensify depressive disorders. Because of these features, abnormal FOH can be classified as a cluster of anxiety disorders (Krawczyk et al., 2020). Moreover, patients with high levels of fear tend to maintain higher glucose levels to prevent hypoglycemia, which may lead to suboptimal metabolic control of diabetes and increase the risk of long‐term complications (Krawczyk et al., 2020). The FOH is manifested as the fear of the negative impact of hypoglycemia on health and life, fear of an emergency, fear of losing self‐control, and the onset of behavioral or cognitive disorders, which may result in behaviors that are generally socially unacceptable (Böhme et al., 2013; Gjerløw et al., 2014). All of this determines the daily functioning of patients. The FOH concerns not only diabetic patients but also their parents, caregivers, partners, and other people from their close environment (Monaghan et al., 2009).

### 2.3 Risk factors for the FOH

This risk of anxiety disorders, including FOH, might be increased by different factors, such as the female sex, genotype (e.g., mutations in the gene coding for monoamine oxidase A [MAO‐A] regulating the catabolism of dopamine, serotonin, and noradrenalin), certain personality types (avoidant, dependent, obsessive‐compulsive personality), and environmental and psychosocial factors (e.g., negative experiences, difficult relationships with family and peers) (Beléndez & Hernández‐Mijares, 2009; Gjerløw et al., 2014; Irvine et al., 1992; Kępiński, 2020). There is also a negative correlation between the intensity of FOH and education level (Gonder‐Frederick et al., 2011). Not only the severity but also the frequency of previous hypoglycemic episodes is related to the intensity of the FOH (Irvine et al., 1992; Polonsky et al., 1992). The experience of an episode of severe hypoglycemia in the last 12 months, as well as the unawareness of hypoglycemia associated with a higher risk of future hypoglycemia, significantly increases the level of fear (Anderbro et al., 2010; Böhme et al., 2013). Moreover, the level of fear increases with the number of symptoms experienced during mild hypoglycemic episodes (Anderbro et al., 2010, 2015). The occurrence of hypoglycemia and fear is higher at night, which deteriorates sleep quality and, for this reason, has a negative effect on the patient's quality of life (Gjerløw et al., 2014; Martyn‐Nemeth, Quinn, Phillips, et al., 2014).

The FOH may determine the behavior of patients and influence their insulin dosing, physical activity, and food intake (Brazeau et al., 2008; McCoy et al., 2013; Zander et al., 2014). When there is an anticipated risk of low blood glucose levels, patients consume additional meals, especially those rich in simple carbohydrates, and tend to snack at night (Desjardins et al., 2014; Richmond, 1996). This behavior is particularly common in women with type 1 diabetes (Martyn‐Nemeth, Quinn, Hacker, et al., 2014). In one study on patients with type 1 diabetes, the mean number of hypoglycemic episodes was 5.7 ± 3.8 per week, which was associated with the intake of an additional 600 kcal per week (Molęda et al., 2017). A high level of FOH is associated with greater differences in blood glucose levels and increased caloric intake and prevents patients from taking up physical activity since they exercise less frequently and less intensively (Brazeau et al., 2008; Martyn‐Nemeth et al., 2017). Another study revealed the highest level of FOH in patients with low mean glucose levels and high glycemic variability (Irvine et al., 1992). There was no clear correlation between metabolic control of diabetes and FOH. On the one hand, greater fear makes patients maintain higher glucose levels and is associated with higher values of glycated hemoglobin (HbA_1c_). On the other hand, a higher level of fear stimulates greater vigilance and motivates patients to more effective glycemic control (Gonder‐Frederick et al., 2011; Hanna et al., 2014; Molęda et al., 2017).

## 3 Measuring levels of FOH

### 3.1 Hypoglycemia Fear Survey

The intensity of fear is a very subjective feeling and is difficult to measure. Different questionnaires have been used for this purpose. The first instrument used to assess levels of FOH was the Hypoglycemia Fear Survey (HFS), developed by Cox et al. and validated for psychometric properties in 1987 (Cox et al., 1987). Currently, it is widely used in a modified version (HFS‐II), which is a 33‐item questionnaire organized into two subscales. The first subscale concerns behaviors aimed at preventing hypoglycemia, while the second subscale concerns worries associated with hypoglycemia and its consequences. The validity of each item in the questionnaire is scored by the patient from 0 (never) to 4 (always) depending on their personal experience from the last 4 weeks. Then, the scores obtained in both subscales are summed. The maximum score is 60 points for the first subscale and 72 points for the second subscale. The higher the total score obtained for the whole questionnaire is, the higher the level of fear in the patient. Both subscales of the survey also can be analyzed separately. The HFS was originally developed for adult patients with type 1 diabetes, but over time, it found its application in patients with type 2 diabetes, pregnant women, and children. HFS‐II also has been translated into and adapted for many languages (Haugstvedt et al., 2010; Jeddou et al., 2017).

### 3.2 Other scales examples

Other tools for measuring the FOH include Quick Screening for Fear of Hypoglycemia (QSFH), the Fear of Hypoglycemia 15‐item scale (FH‐15), and the Children's Hypoglycemia Index (CHI) used in pediatric patients (Anarte Ortiz et al., 2011; Kamps et al., 2005; Schmidt et al., 2017). The introduction of screening for FOH in the standards of diabetes care is critical, as it highlights the problem faced by patients with diabetes. Screening for FOH is aimed at ensuring the patient's better quality of life, improving metabolic control of diabetes, and the effects of treatment. Identification of patients at particularly high risk of developing abnormal fear enables the early provision of specialist support and implementation of therapeutic measures. Studies have demonstrated that physicians often have limited knowledge about their patients, and patients also are reluctant to share their experiences related to the FOH (Böhme et al., 2013).

## 4 Therapies for the FOH

Patient education plays an important role in reducing the FOH. Patients who are aware of the dangers associated with hypoglycemia can reduce the frequency of hypoglycemic episodes and eliminate severe hypoglycemia by adequate management and self‐control, thus modifying the most important factors influencing the development of fear (Bhutani et al., 2015). Psychotherapy plays the most important role in the treatment of anxiety disorders. Early identification of patients with abnormal levels of FOH allows for prompt implementation of adequate measures. It has been proven that cognitive behavior therapy has positive effects in subjects with abnormal FOH. A psychotherapeutic intervention relying on this approach with a subsequent maintenance period allowed for better metabolic control of diabetes measured based on HbA_1c_ values eight, 24 and 48 weeks after the end of therapy. Moreover, reduced levels of fear on the behavioral subscale, depressive symptoms, and increased scores for well‐being were observed in patients using this therapy compared to the control group (Amsberg et al., 2009; Anderbro, 2012).

Psychoeducational interventions targeted at hypoglycemia, such as hypoglycemia anticipation, awareness and treatment training (HAATT), blood glucose awareness training II (BGAT‐2), and the hypoglycemia treatment program (HyPOS), have been developed worldwide. The aim of these programs is to teach patients daily self‐observation and self‐control (e.g., how to keep a hypoglycemia diary) combined with group meetings targeted at educating patients on proper diet, physical activity, insulin therapy, or management of acute complications of diabetes, especially ketoacidosis. Studies have shown that participants in these programs responded earlier and more appropriately to low glucose levels, which reduced the number of hypoglycemic episodes (Cox et al., 2001, 2004; Hermanns et al., 2007). Moreover, BGAT‐2 training contributed to a reduction of glycemic variability, a reduction of the intensity of FOH and depressive symptoms, and an improved quality of life for the participating patients with type 1 diabetes (Cox et al., 2001). On the other hand, HypoCOMPaSS, aimed at patients with hypoglycemia unawareness, emphasizes the prevention of hypoglycemia, strict control of blood glucose levels and identification of circumstances that could lead to a drop in glucose levels. Patients treated with intensive insulin therapy used an application to calculate their insulin dose (bolus calculator) while simultaneously using the continuous glucose monitoring system or taking self‐measurements of blood glucose with a glucose meter. In all patients, a significant reduction in the frequency of hypoglycemia, intensity of FOH, and improved awareness of hypoglycemia and glucose variability were observed, and 24 weeks after the end of the training, the patients still controlled their glucose levels more effectively than before the study (Little et al., 2014).

Psychoeducational programs are aimed not only at patients but also people from their close environment. Reducing Emotional Distress for Childhood Hypoglycemia in Parents (REDCHiP) is a program that includes seven group sessions and three individual sessions conducted using telemedicine techniques. Participating parents of children with type 1 diabetes were re‐educated about the disease affecting their children and learned about methods of cognitive and behavioral therapy. Studies using REDCHiP have shown a significant reduction in the levels of FOH and stress in parents (Marker et al., 2020; Patton et al., 2020).

## 5 Modern technologies and FOH

New devices used by diabetic patients allow for more accurate glycemic monitoring and better metabolic control of diabetes (Wunna et al., 2021). Their use in the management of diabetes may help reduce the FOH. Sensor‐augmented personal insulin pumps offer the continuous monitoring of blood glucose levels, visualization of decreasing blood glucose trends, setting alerts at specific blood glucose levels, and an automatic low glucose suspend function (LGS). These functions are aimed at preventing the occurrence of hypoglycemia. On the other hand, continuous glucose monitoring systems (CGMS) allow for the control of asymptomatic hypoglycemia, especially during sleep, thanks to which it is possible to adjust the insulin dose and prevent further hypoglycemic episodes.

### 5.1 Continuous glucose monitoring systems

Continuous glucose monitoring systems constantly track blood glucose concentrations and read them in real time. A randomized controlled trial by the Juvenile Diabetes Research Foundation compared patients with standard blood glucose measurements taken with those using a CGMS. Reduced FOH was found in the behavioral subscale of the HFS‐II questionnaire in the CGMS group after 26 weeks of follow‐up (Beck et al., 2010). In a study by Halford et al. aimed at determining the clinical and psychological benefits of continuous glucose monitoring, patients reported a reduction in fear while using a CGMS (Halford & Harris, 2010). However, in patients with suspected anxiety disorders, caution is advised when using CGMSs, because paradoxically, the constant control of glucose levels may increase their anxiety and lead to withdrawal from therapy. The patient's constant compulsion to check their blood glucose level and the accompanying need to take action, often too early and for no reason, may result in a persistent feeling of tension and anxiety, resulting in a reduction in the quality of life and the cessation of CGMS use (O'Donnell et al., 2019).

### 5.2 Flash glucose monitoring systems

The flash glucose monitoring system (FGMS) consists of a sensor that continuously measures interstitial glucose level and a reader device. After swiping the reader device over the sensor, a patient obtains his/her current interstitial glucose level and 8‐h trend graph. FGMS allows to demonstrate prolonged higher glucose levels after meals and night hypoglycemia episodes that a patient is often unaware of. The 18‐month study trial by Rouhard et al. indicates that using FGMS in patients with type 1 diabetes may not only improve glycemic control but also reduce FOH in the behavioral subscale of the HFS‐II questionnaire (Rouhard et al., 2020).

### 5.3 Bolus calculator

The bolus calculator is a function of an insulin pump, glucose meter, or a mobile application designed to precisely calculate the insulin dose required in a given situation, appropriate to the amount of carbohydrates taken in a meal and adjusted to the current glucose level and the amount of active insulin still circulating in the body. Studies on patients with type 1 diabetes treated with multiple insulin injections showed that the use of an automatic bolus calculator was associated with reduced FOH (Barnard et al., 2012; Vallejo Mora et al., 2017).

### 5.4 Personal insulin pump

The use of a personal insulin pump in the treatment of diabetes reduces the number of hypoglycemic episodes, especially severe episodes (Quirós et al., 2016). Studies also have revealed that patients treated with a personal insulin pump are characterized by a lower FOH measured by the behavioral subscale than patients using multiple insulin injections with pens (Barnard & Skinner, 2008). The STAR 3 randomized clinical trial compared the metabolic control of diabetes in patients treated with a sensor‐augmented personal insulin pump and the metabolic control of patients using multiple insulin pen injections with self‐measured blood glucose. Patients’ quality of life and satisfaction with treatment were also considered in this trial, including the FOH. After a 12‐month follow‐up, improved hypoglycemic behavior scores and a reduced level of worry were found in patients treated with a sensor‐augmented personal insulin pump (Rubin & Peyrot, 2012).

### 5.5 Closed loop system

A closed loop system, also known as an artificial pancreas, aims to deliver insulin in the most physiological way possible. It consists of an insulin pump and a sensor that continuously measures blood glucose coordinated by an algorithm that allows insulin delivery in response to changes in blood glucose levels. In a study investigating the effect of an artificial pancreas on the level of FOH, the 4‐day night time use of this system was associated with reduced fear and high treatment satisfaction in patients with type 1 diabetes (Ziegler et al., 2015).

### 5.6 Low glucose suspension function

The low glucose suspension function automatically suspends insulin delivery by the personal insulin pump at low blood glucose levels. In the CGM TIME trial, which involved 144 children with type 1 diabetes for at least 1 year, patients were treated using a sensor‐augmented personal insulin pump with the LGS function, and parents completed the HFS‐II questionnaire at baseline and after 12 months of treatment. The study demonstrated that personal insulin pump therapy with the LGS function significantly reduced the FOH (Verbeeten et al., 2021). Another technology used in sensor‐augmented personal insulin pumps predicts drops in glucose levels 30 min before reaching a given value and allows insulin delivery to be automatically suspended and then automatically continued when the downward trend in blood glucose has subsided. A study on 21 children with type 1 diabetes investigating the effectiveness of this system for the prevention of hypoglycemia revealed that this function helped reduce the risk of hypoglycemia without a significant negative effect on the metabolic control of diabetes or an increased incidence of ketoacidosis (Villafuerte Quispe et al., 2017).

## 6 Clinical examples of abnormal FOH in patients

Identification of patients with a high FOH is possible based on the patient's history, assessment of metabolic control, and self‐management of the patient with diabetes. Taking the HFS‐II questionnaire also plays an important but secondary role in identifying this problem. Two cases of patients with type 1 diabetes with a history and glycemic profile characteristic of maladaptive FOH are presented below. The high FOH in one patient was manifested by different behaviors, such as reducing the insulin doses recommended by the bolus calculator or stopping the pump when the sensor‐detected or self‐measured glucose levels showed a declining trend, even if the blood glucose level was still high.

**FIGURE 1:**
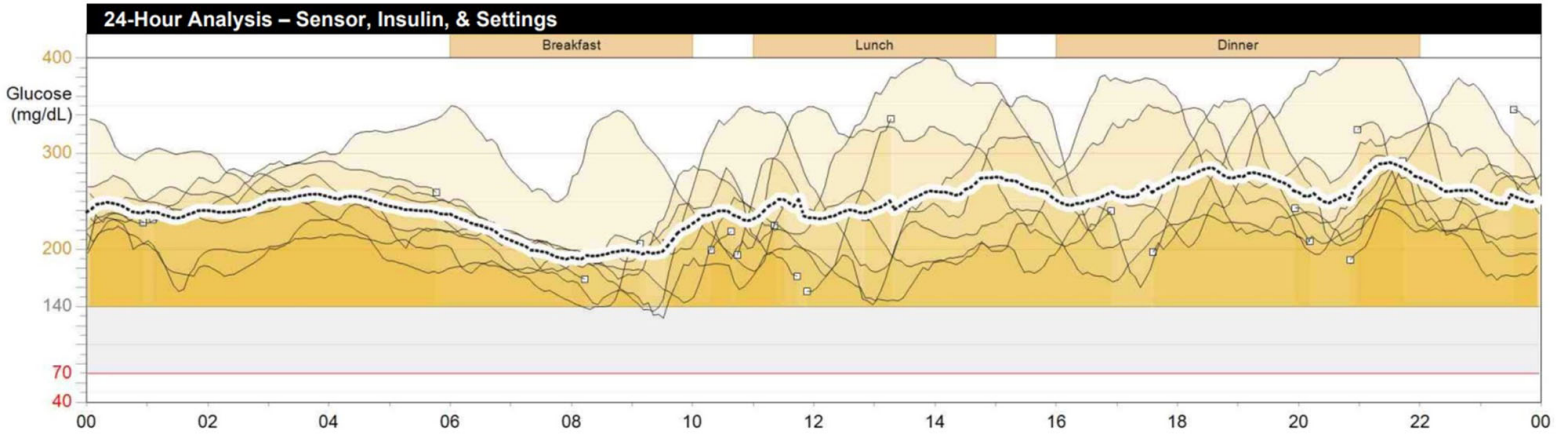
Records from the personal insulin pump of a 22‐year‐old pregnant patient. It is a 24‐h analysis of glucose levels from 7 days. Almost all of records are above the recommended glucose level. Further description in the text

Figure 1 shows records from the personal insulin pump of a 22‐year‐old pregnant woman suffering from type 1 diabetes for 15 years who was characterized by a very high FOH. In the HFS‐II questionnaire, her score was 31 on the behavior subscale and 59 on the worry subscale. The patient had a history of four episodes of severe hypoglycemia with loss of consciousness. The current HbA_1c_ value, measured in a lab test at the time of reading the PIP records and completing the HFS‐II questionnaire, was 85 mmol/mol (9.9%). The report for 2 weeks of PIP operation showed an average blood glucose level of 12.7 ± 3.8 mmol/L (228 ± 68 mg/dl), with 7.2 daily blood glucose measurements. For almost 100% of that time, the patient's glucose level was above the recommended level (<7.8 mmol/L [<140 mg/dl]). Moreover, the woman prefers having high glucose levels despite the fact that it could have dangerous consequences for the growth and functioning of her baby.

**FIGURE 2:**
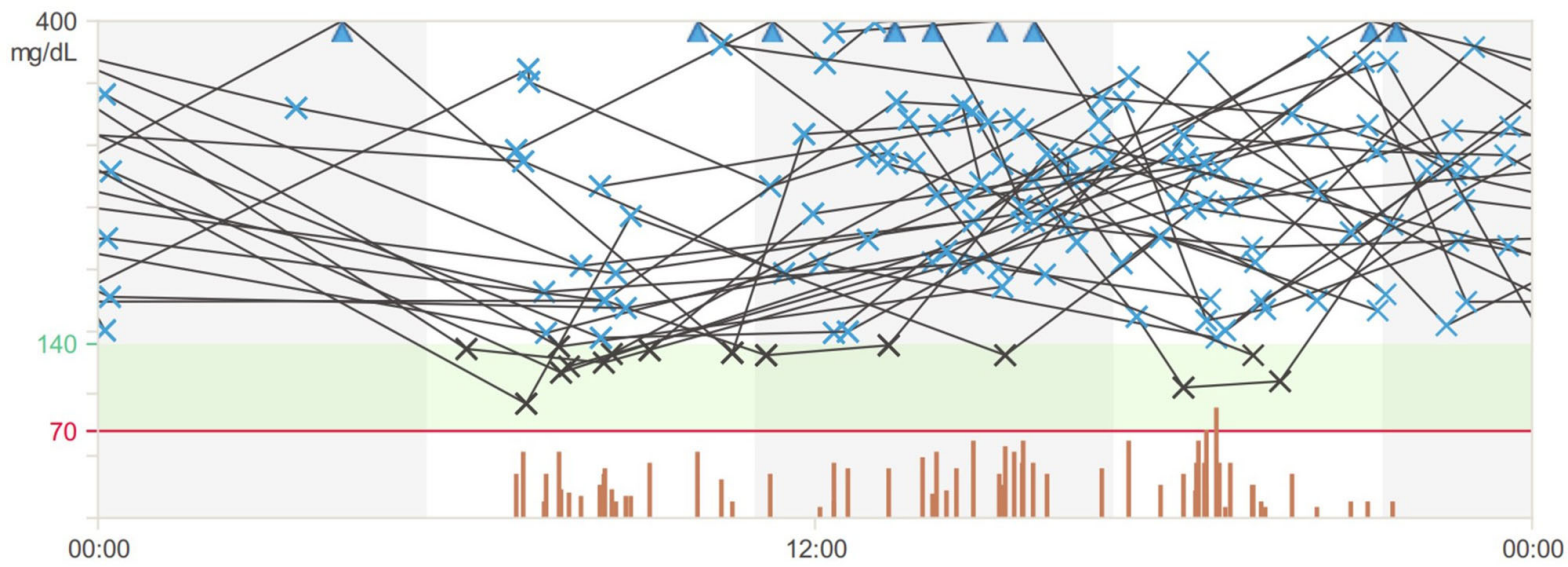
Records from the glucose meter of a 26‐year‐old female patient. It is a 24‐h analysis of glucose levels from 14 days. Majority of records are above the recommended glucose level. Further description in the text

Figure 2 presents records from the glucose meter for a 26‐year‐old woman suffering from type 1 diabetes for 15 years who also had a high FOH. In the HFS‐II questionnaire, her score was 32 on the behavior subscale and 34 on the worry subscale. During the analyzed month, the mean number of blood glucose measurements was 5.3 per day, the mean blood glucose level was 14.3 ± 4.8 mmol/L (258 ± 87 mg/dl), and 90% of the measurements were above the target range (3.9–7.8 mmol/L [70–140 mg/dl]). The patient did not experience any episode of hypoglycemia during the analyzed period of time.

The described patients had high levels of fear, which was reflected in their behaviors aimed at maintaining high glucose levels and preventing the proper metabolic control of diabetes. Both women were unable to meet the glycated hemoglobin target values or the time spent at the glycemic target, despite the use of advanced devices.

## 7 CONCLUSIONS

Adaptive FOH in a patient suffering from diabetes has positive aspects and allows for a response appropriate to the risk of hypoglycemia. On the other hand, abnormal fear causing constant tension, anxiety, restriction of freedom, and reduced quality of life is pathological and should be identified early enough by the caregivers of patients with diabetes. The widespread use of hypoglycemia fear surveys in clinical practice to assess the level of fear during a visit to a diabetologist could improve the quality of diabetes care. Equally important is the competent and early identification of patients at risk of developing abnormal fear by analyzing the patient's history and treatment outcomes. An important issue that can prevent hypoglycemia, ensure good metabolic control and adequate self‐control is ongoing patient education by qualified personnel, including with the use of psychoeducational training designed for this specific purpose. Advanced therapeutic technologies, such as continuous glucose monitoring, personal insulin pumps, the bolus calculator function, and the low glucose suspended function, especially when combined, also may help reduce the FOH. Offering participation in individual or group psychotherapeutic sessions aimed at cognitive‐behavioral therapy to patients with FOH who meet the criteria for anxiety disorders at an appropriately early time may help reduce the level of fear and improve the quality of life of these patients. Consideration of FOH is a vital aspect of diabetes care because the presence and level of this fear determine the type of therapy and management of patients with diabetes. The priority areas are to popularize knowledge about the FOH and its consequences among people who have direct contact with diabetes patients, as well as to conduct further studies to identify factors that determine the level of fear, which can be useful in planning personalized therapies for these patients.

## CONFLICT OF INTEREST

The authors declare no conflict of interest.

### PEER REVIEW

The peer review history for this article is available at https://publons.com/publon/10.1002/brb3.2633.

## Data Availability

Data sharing not applicable to this article as no datasets were generated or analyzed during the current study.
